# Supplementary material for: The causal relationship between the human gut microbiota and pyogenic arthritis: a Mendelian randomization study
Source: Front Cell Infect Microbiol. 2024 Nov 26;14:1452480. doi: 10.3389/fcimb.2024.1452480 (PMC11629706; doi:10.3389/fcimb.2024.1452480)
Supplement: Supplementary file 1 [file DataSheet1.zip › mendelian test/ebi-a-GCST90027500.csv_forest.pdf]

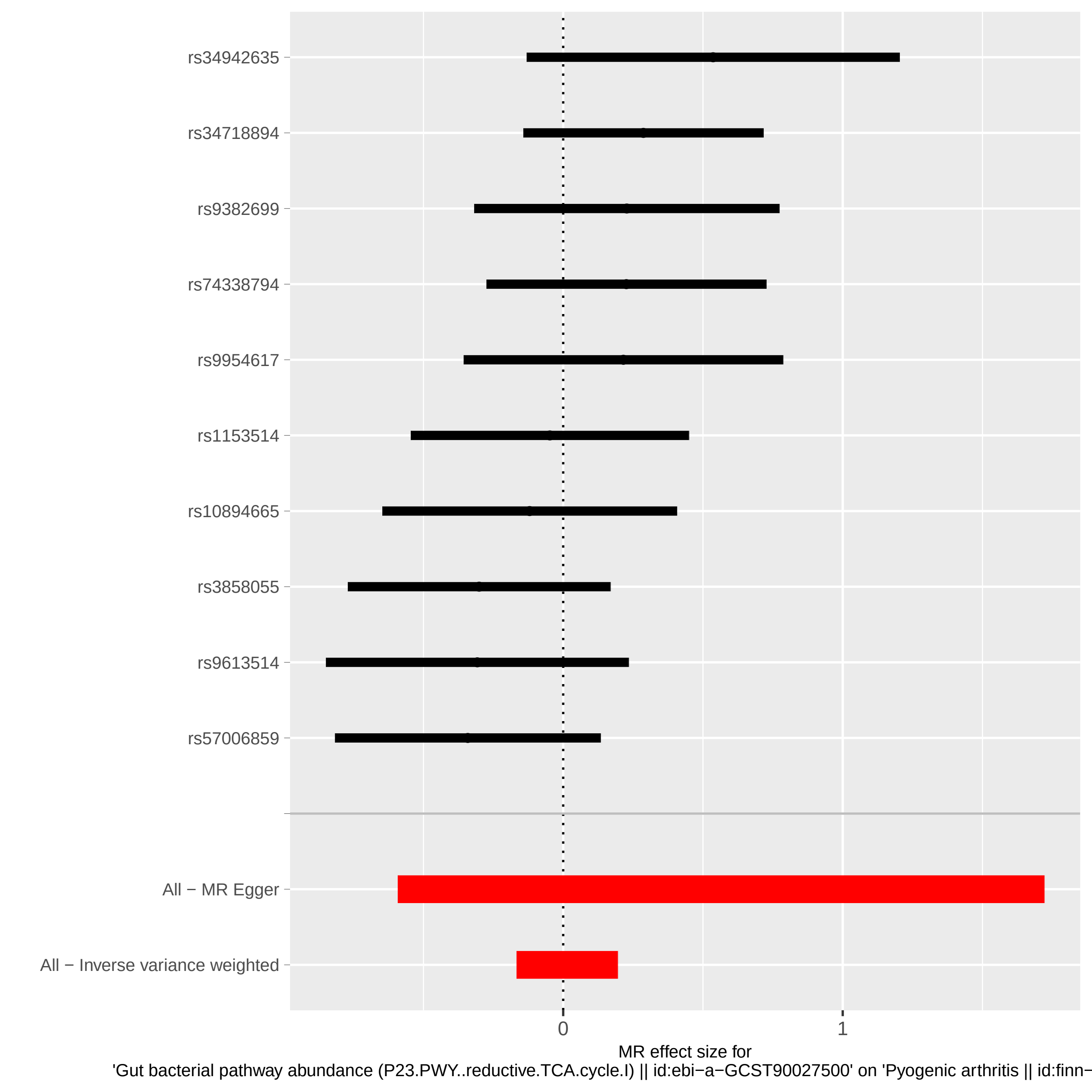

rs34942635
rs34718894
rs9382699
rs74338794
rs9954617
rs1153514
rs10894665
rs3858055
rs9613514
rs57006859
All − MR Egger
All − Inverse variance weighted
0
1
MR effect size for
'Gut bacterial pathway abundance (P23.PWY..reductive.TCA.cycle.I) || id:ebi−a−GCST90027500' on 'Pyogenic arthritis || id:finn−
